# Measuring Recovery Using the Individual Recovery Outcomes Counter: A Cross‐Sectional Multi‐Center Study on Structural Validity in Dutch Mental Health Care

**DOI:** 10.1111/inm.70093

**Published:** 2025-07-24

**Authors:** Margot Metz, Gabriëlle van Son, Floor Stuit, Nelleke van der Weerd, Erik de Groot, Edwin de Beurs

**Affiliations:** ^1^ GGz Breburg Mental Health Care Organization Tilburg the Netherlands; ^2^ Tranzo Scientific Center for Care and Wellbeing Tilburg University Tilburg the Netherlands; ^3^ GGZ Rivierduinen Mental Health Care Organization Leiden the Netherlands; ^4^ SynQuest Cooperation of Mental Health Care Organizations Leiden the Netherlands; ^5^ Amsterdam UMC Amsterdam the Netherlands; ^6^ GGZ InGeest Mental Health Care Organization Amsterdam the Netherlands; ^7^ Dimence Group Mental Health Care Organization Deventer the Netherlands; ^8^ Arkin Mental Health Care Organization Amsterdam the Netherlands; ^9^ Leiden University Leiden the Netherlands

**Keywords:** factor analysis, I.ROC, mental health care, recovery, structural validity

## Abstract

The Individual Recovery Outcomes Counter (I.ROC) is a recovery orientated measure, originating from Scotland, which is increasingly used in Dutch mental health care. The aim of this study is to extend previous research into the structural validity of the I.ROC. We investigated the factor structure of the Dutch I.ROC among mental health care clients treated in various settings using data (*N* = 8635) from five Dutch mental health care organisations. We conducted an exploratory factor analysis (EFA, *N* = 4295) and confirmatory factor analysis (CFA, *N* = 4340), and tested the fit of factor structures found in previous research. EFA revealed support for both a one‐ and a two‐factor structure: ‘Total‐scale’ (12 items; α = 0.88), ‘Empowerment’ (8 items; *α* = 0.86) and ‘Vitality and Activity’ (4 items; α = 0.67). CFA indicated a good fit for a modified two‐factor model found in previous research on a representative sample of the Dutch population comprising ‘Wellbeing, control, network and meaningfulness’ and ‘Health safety and abilities’ (CFI = 0.944). Furthermore, the two‐factor solution of this study showed a good fit (CFI = 0.940). However, these findings were not conclusive, as the one and alternative two‐, three‐, or four‐factor models of other studies also demonstrated an acceptable fit. For use with individual patients, inspection of scores on individual items (in a spider graph) is most useful. As proven in several studies, the one‐factor structure can be used for summarisation. Additionally, multiple solutions for subscale scores proved to be a good fit. Overall, the structural validity of the I.ROC requires further investigation and research with longitudinal data is recommended.

## 1 Introduction

Traditionally, the leading paradigm in mental health care was based on the medical model, which assumed that each illness has a specific biological cause and its own symptomatic treatment (Dean 2017). For mental health care, this led to a dominant practitioner‐oriented view, with a strong emphasis on symptoms, diagnosis and a search for ways to objectify outcomes with instruments in which this view was also incorporated. However, several authors noticed differences between the view of the consumers and the practitioners concerning recovery and outcome in (severe) mental illnesses (Davidson et al. 2008; Jose et al. 2015). Under the influence of the mental health service user movement, an additional understanding of recovery emerged (Slade 2010; van Weeghel et al. 2019; Dell et al. 2021). Instead of merely pursuing a symptom‐free state, nowadays recovery is also seen as a strength‐oriented process where the person's own perspective, who is the expert on their own recovery journey, is important (Slade and Longden 2015). The focus lies more on promoting the clients' recovery in various areas of life in line with the clients' wishes (Boevink 2012; Jose et al. 2015). In recovery oriented care the concept of recovery implies that clients can lead a life as meaningful and satisfying as possible despite the (persistent) presence of a mental illness (van Weeghel et al. 2019). A view which is in line with the more adaptive current view on health as defined by the WHO “Health as the ability to adapt and self‐manage in the face of social, physical and emotional challenges” (Huber et al. 2011; van Druten et al. 2022). Reviews on recovery in mental health confirms the importance of a more holistic and personalised approach in which social factors such as safety, housing situation and income, in addition to psychological factors, have a major influence on psychological well‐being (van Weeghel et al. 2019; Llewellyn‐Beardsley et al. 2019; Dell et al. 2021).

With the emergence of the recovery‐oriented view, there is a need in the mental health sector for reliable and validated instruments to properly measure clients' progress in recovery. The Individual Recovery Outcomes Counter (I.ROC) (Ion et al. 2013; Monger et al. 2013) is an example of a recovery orientated measure which is increasingly used in Dutch mental health care organisations. The I.ROC is an instrument for identifying treatment goals in various areas of life and well suited for monitoring progress towards those goals. The I.ROC consists of 12 items scored on a six‐point scale form 1 ‘never’ to 6 ‘all the time’. Higher scores represent more wellbeing and recovery. Clients and practitioners are positive about the instrument as it is easy to use and has an attractive visual design (Ion et al. 2013; Monger et al. 2013). Using themes for various life areas, clinical, social and personal recovery is represented from the client's perspective. The instrument can be applied as a self‐report measure, to be completed by the patient, or as a guide in an interview by a practitioner to explore the client perspective on life areas (Baufeldt and Dawson 2022; Monger et al. 2013). Initially, the I.ROC was designed to help the client and practitioner to set and evaluate recovery goals in a collaborative decision‐making process (Metz et al. 2023). In addition, the questionnaire can also be used by teams to learn from I.ROC outcomes at group level, to get a picture of how clients score on the various items (life areas) and how these scores change during treatment (Sportel et al. 2023).

The I.ROC was developed by Penumbra, a mental health care provider in Scotland where the initial validation research took place (Monger et al. 2013; Dickens et al. 2017; Rudd et al. 2020). This was followed by the Dutch translation and validation of the I.ROC in the Netherlands (Beckers et al. 2022; Roze et al. 2019; Sportel et al. 2023), where the I.ROC is increasingly being used in a broad mental health population, both among clients with Severe Mental Illness (SMI) and non‐SMI. A Dutch consensus group (Delespaul 2013) described SMI as the group of mental health clients with severe long‐term impairments (at least several years) in functioning, which are both cause and effect of the psychiatric disorder and require coordinated care.

The validation studies in Scotland and the Netherlands showed positive results regarding the reliability and validity of the English and Dutch versions of the I.ROC. However, the structural validity (factorial structure) is still debated, as I.ROC studies in various populations in the mental health and general population showed a diversity of factor structures, which are reported in detail in Table 1. Initially, a theoretical and visual framework of four dimensions were proposed, visualised as quadrants in a radar plot, called the HOPE framework (Home Opportunity, Empowerment and People) (Ion et al. 2013). Next, in Scotland Dickens et al. (2017) reported a unidimensional structure, Monger et al. (2013) a two‐dimensional structure (labelled as ‘interpersonal’ versus ‘intrapersonal’) and Rudd et al. (2020) a three‐dimensional factor structure (allocating 10 of the 12 items to the following concepts: Psychological wellbeing and health, Decision‐making and life skills and Meaningful activity). Originally, in the Netherlands the factor structure was mainly studied with clients suffering from severe mental illness (SMI) (Beckers et al. 2022; Sportel et al. 2023). Dutch research by Sportel et al. (2023) examined various factor structures in clients diagnosed with psychosis and found no differences in ‘best fit’ for the one, two or four dimensional models. Lastly, a recent analysis of the factor structure in a representative Dutch sample of the general population found a two‐factor solution (van Druten et al. 2024).

**TABLE 1:** Factor structures using all the 12‐items: Results of previous research.

Study	Type of FA	Factors	Results
Monger et al. (2013)	EFA (no further information)	F1 = 1–4, 7, 9–11 (intrapersonal) F2 = 5, 6, 8, 12 (interpersonal)	51.8% explained variance; no further information provided
van Druten et al. (2024)	EFA—PCA with oblimin rotation	F1 = 1, 6–12 (wellbeing, control, network, and meaningfulness) F2 = 2–5 (health, safety, and abilities)	56.1% explained variance, no further information
Dickens et al. (2017)	CFA	F1 = 1–3 F2 = 4–6 F3 = 7–9 F4 = 10–12 (HOPE model)	χ^2^(48) = 362.062; CFI = 0.95; NFI = 0.93; GFI = 0.97; AGFI = 0.95; RMSEA = 0.061 [0.056–0.067]
	CFA	F1 = 4–6, 8 F2 = 1–3, 7, 9–12	χ^2^(48) = 90.93; CFI = 0.95; NFI = 0.93; GFI = 0.97; AGFI = 0.95; RMSEA = 0.061 [0.056–0.067]
Rudd et al. (2020)	CFA	Single factor	χ^2^(54) = 96.84; χ^2^/1.79; CFI = 0.84; IFI = 0.85; SRMR = 0.08; RMSEA = 0.08 [0.058–0.114]
		1–3 4–6 7–9 10–12 (HOPE model)	χ^2^(48) = 90.93; χ^2^/1.89; CFI = 0.84; IFI = 0.85; SRMR = 0.09; RMSEA = 0.09 [0.062–0.120]
		F1 = 1–4, 7, 9–11 (intrapersonal) F2 = 5, 6, 8, 12 (interpersonal) (Monger et al. 2013)	χ^2^(53) = 95.38; χ^2^/1.80; CFI = 0.84; IFI = 0.84; SRMR = 0.08; RMSEA = 0.09 [0.058–0.115]
		F1 = 1–3, 7, 9–12 F2 = 4–6, 8 (Dickens et al. 2017)	χ^2^(53) = 91.54; χ^2^/1.73; CFI = 0.85; IFI = 0.86; SRMR = 0.08; RMSEA = 0.08 [0.053–0.111]
Beckers et al. (2022)	EFA (no rotation)	Single factor	All loadings > 0.50
Sportel et al. (2023)	CFA	Single factor	CFI = 0.912, TLI = 0.892; RMSEA = 0.088
		F1 = 1–3 F2 = 4–6 F3 = 7–9 F4 = 10–12 (HOPE model)	CFI = 0.913, TLI = 0.881; RMSEA = 0.092
		F1 = 1–4, 7, 9–11 (intrapersonal) F2 = 5, 6, 8, 12 (interpersonal)	CFI = 0.915, TLI = 0.894; RMSEA = 0.087
van Druten et al. (2024)	CFA‐MLE Maximum Likelihood Estimation	F1 = 1, 6–12 (wellbeing, control, network, and meaningfulness) F2 = 2–5 (health, safety, and abilities)	χ^2^ (47) = 321.50; CFI = 0.96; RMSEA = 0.068 [0.061–0.075]

Following previous research results, for clinical practice it is relevant to further substantiate the structural validity of the I.ROC aiming to explore whether a framework can be recognised that best fits with data from a broad mental health population, including SMI and non‐SMI as well. To address this theme, we investigated the factor structure among mental health care clients treated in different settings using data from five mental health care organisations across the Netherlands. A large dataset was split into two halves. We conducted an exploratory factor analysis (EFA) on the first half, followed by a confirmatory factor analysis (CFA) on the second half to test the fit of the found EFA structure. Additionally, we investigated the fit of factor structures found in previous research and we tested if the factor structures differ between the SMI and non‐SMI group.

## 2 Methods

### 2.1 Study Design

An observational cross‐sectional research design was used to evaluate the factor structures of the I.ROC in a Dutch mental health population. We conducted an exploratory factor analysis on a random selection of 50% of our sample, followed by a confirmatory factor analysis on the other half aimed to test the fit of the exploratory factor structure. Additionally, we investigated the fit of frameworks found in previous research.

### 2.2 Participants

Five Dutch mental health care organisations participated in this study. We distinguished two subgroups in this study, that is, adults (age ≥ 18 years) with Severe Mental Illness (SMI) and non‐Severe Mental Illness (non‐SMI).

Based on the definition of SMI (Delespaul 2013) we classified clients by treatment setting in SMI and non‐SMI. Clients in treatment by Flexible Assertive Community Treatment (FACT), Active Recovery Triad (ART), Early Intervention Psychosis (EIP) and basic chronic mental health care (bggz chronic) teams were classified in the SMI group. Clients treated by outpatient teams specialised in depression, anxiety, personality, and neurodevelopmental disorders (i.e., autism spectrum disorder, attention deficit hyperactivity disorder) were divided into the non‐SMI group. In a limited number of wards, both SMI and non‐SMI patients were treated. As the distinction for these groups could not be reliably made, these data were not included in the additional analyses for the SMI and non‐SMI group.

### 2.3 Data Collection

The I.ROC questionnaire was administered routinely as part of the treatment. The first available I.ROC measurements at the start of treatment were used in the analyses. First measurements were included if completed within 3 months (< 93 days) after registration at the participating mental health care organisation. Some of the clients could have been previously in treatment in the same or other mental health care organisations. Before sending the data to the researchers (FS, EdB), the five participating mental health care organisations all anonymised the data according to agreed rules based on the General Data Protection Regulation (GDPR). In addition we conformed to the internal procedures of the participating mental health organisations, which meant that we did not use data from clients who objected to the anonymous use of data for scientific research.

To perform factor analyses, rules of thumb for sample size calculation vary from 4 to 10 clients per item with a minimum of 100 clients (de Vet et al. 2011). With 8635 participants and 12 I.ROC items, this study comfortably meets this standard.

### 2.4 Exploratory Factor Analysis

To explore whether a framework in the I.ROC can be recognised that best fits with data from a broad Dutch mental health population, exploratory factor analysis was employed, adhering to the following steps (de Vet et al. 2011) for the total sample as well as for the subgroups Severe Mental Illness and non‐Severe Mental Illness separately:

- Step 1: Associations between all items were assessed with Kaiser‐Meyer‐Olkin (KMO) and Bartlett's test correlation in total data; these should be *r* > 0.5 with a significance level of *p* < 0.05 to have sufficient correlations for factor analysis.
- Step 2: The number of factors was determined with a criterion eigenvalue > 1 and inspection of the scree plot for confirmation. Multiple analyses were conducted, first by setting the number of factors to be extracted as all those with eigenvalues greater than one and, secondly, analyses were run with the number of extracted factors manually set equal to the number of factors found in previous analyses (Monger et al. 2013; Dickens et al. 2017). The interpretability of the factor solution was improved with Oblimin rotation as items and factors were expected to be correlated. Factor loadings of the rotated factors are presented, omitting loadings < 0.30 (de Vet et al. 2011). Items were allocated to factors based on their highest factor loading. Factor loadings of > 0.71 are considered excellent, > 0.63 very good, > 0.55 good and > 0.45 fair (Tabachnick and Fidell 2007).
- Step 3: Item content and factor structure were discussed by the research team. If items loaded equally on multiple factors, the item was assigned to the factor that fitted best conceptually, according to the research team.
- Step 4: Inter‐item correlations per factor were assessed; values should be between *r* = 0.2 and 0.5. Inter‐item correlations greater than *r* = 0.7 suggest that they may measure the same concept and are redundant (de Vet et al. 2011).
- Step 5: Cronbach's alphas were calculated to establish internal consistency of the resulting factors; values of alpha between 0.7 and 0.9 for factors with a minimum of 3 items were considered good (de Vet et al. 2011).
- Step 6: In order to label the different factors, the item content and factor loadings were discussed again among the research team.

EFA analyses were conducted using SPSS version 29.

### 2.5 Confirmatory Factor Analysis

To test the factor model that arose from analysing the first half of the data in the present study with exploratory factor analysis, a confirmatory factor analysis (CFA) was conducted. In a secondary analysis, we made the distinction between the subgroups Severe Mental Illness and non‐Severe Mental Illness.

Additionally, we tested with confirmatory factor analyses whether previously found factor structure models for the 12 items of the I.ROC (see Table 1) fitted for our sample.

All CFA analyses were done with the R package Lavaan. Fit indices were evaluated according to the following values for indices: CFI/NFI/GFI > 0.95, SRMR/RMSEA < 0.05 (Hu and Bentler 1999; Byrne 2013).

## 3 Results

### 3.1 Descriptives

Participants were 8635 adults newly registered in Dutch mental health care. Descriptives of the sample size are shown in Tables 2 and 3. From the total sample, 70.1% belong to the subgroup non‐SMI and 19.7% to the subgroup SMI. 10.2% could not be classified in one of these subgroups. These data were only included in the analyses of the total sample.

**TABLE 2:** Descriptives of organisation, setting, splitted EFA/CFA groups (*N* = 8635).

	*N* (%)
Organisation	
GGz Breburg	4147 (48.0)
GGZ Delfland	293 (3.4)
Dimence groep	683 (7.9)
Arkin	313 (3.6)
GGZ InGeest	3199 (37.1)
EFA/CFA total sample	
EFA	4295 (49.7)
CFA	4340 (50.3)
Subgroups^a^	
SMI	1701 (19.7)
Non‐SMI	6056 (70.1)
Cannot be classified	878 (10.2)
EFA/CFA subgroups^a^	
EFA SMI	858 (9.9)
EFA non‐SMI	3011 (34.9)
EFA not‐classified	426 (4.9)
CFA SMI	843 (9.8)
CFA non‐SMI	3045 (35.3)
CFA not‐classified	452 (5.2)
Total	8635 (100)

^a^ *N*'s in various analyses diverge due to missing values on items.

**TABLE 3:** Descriptives of the sample total, SMI, non‐SMI, EFA, CFA.

	Total	SMI	non‐SMI	EFA	CFA
	Mean (SD)				
Age	41.6 (15.9)	40.2 (14.2)	42.1 (16.7)	41.6 (16.1)	41.7 (15.7)
Age group	*N* (%)				
12–26	1565 (18.8)	291 (17.1)	1131 (18.7)	821 (19.1)	744 (17.1)
27–31	1276 (15.3)	273 (16.0)	870 (14.4)	620 (14.4)	656 (15.1)
32–38	1395 (16.7)	318 (18.7)	923 (15.2)	685 (15.9)	710 (16.4)
39–48	1416 (17.0)	309 (18.2)	936 (15.5)	689 (16.0)	727 (16.8)
49–59	1377 (16.5)	275 (16.2)	930 (15.4)	677 (15.8)	700 (16.1)
60+	1309 (15.7)	200 (11.8)	1008 (16.6)	660 (15.4)	649 (15.0)
Gender (female)	5013 (60.1)	866 (50.9)	3683 (60.8)	2526 (58.8)	2487 (57.3)
Sub‐total	8338 (96.6)	1666 (97.9)	5798 (95.7)	4152 (96.7)	4186 (96.5)
Missing	297 (3.4)	35 (2.1)	258 (4.3)	143 (3.3)	154 (3.5)
Total	8635 (100)	1701	6056	4295	4340

60.1% of the respondents from the total sample were female and the mean age of the participants was 41.6 years. From 297 respondents, the descriptive age or gender was not known. Table 3 also presents the variables age and gender from the subgroup settings SMI/non‐SMI and EFA/CFA, which were in balance between the subgroups.

The scores of the 12 I.ROC items are shown in Table 4. The mean scores ranged from 2.76 (item 1) to 4.13 (item 2). The values of skewness and kurtosis score within‐1 and 1 indicate a normal distribution.

**TABLE 4:** Basic psychometrics of I.ROC items (*N* = 8635).

I.ROC items	Mean	SD	Skewness	Kurtosis	% Endorsed response					
					1	2	3	4	5	6
1. Mental health	2.76	1.14	0.55	0.09	32	34	14	6	2	12
2. Life skills	4.13	1.30	−0.19	−0.82	9	24	23	25	18	2
3. Satefy and comfort	4.03	1.40	−0.22	−0.86	11	23	21	23	18	4
4. Physical health	3.21	1.32	0.23	−0.66	23	29	20	14	5	9
5. Exercise and activity	3.47	1.45	0.09	−0.87	18	25	22	15	11	9
6. Purpose and direction	3.38	1.37	0.14	−0.71	19	28	22	15	7	9
7. Personal network	3.91	1.36	−0.11	−0.79	11	26	23	21	15	4
8. Social network	2.84	1.38	0.44	−0.54	24	26	17	8	4	20
9. Valuing myself	2.94	1.30	0.51	−0.23	27	33	15	8	5	13
10. Participation and control	3.61	1.30	0.10	−0.64	14	32	23	18	9	5
11. Self management	3.23	1.33	0.31	−0.56	22	32	18	13	6	9
12. Hope for the future	3.01	1.29	0.44	−0.32	25	33	16	9	5	12

### 3.2 Exploratory Factor Analysis

We investigated the factor structure in the randomly selected first half of the data (sample *N* = 4295) with EFA.

**FIGURE 1:**
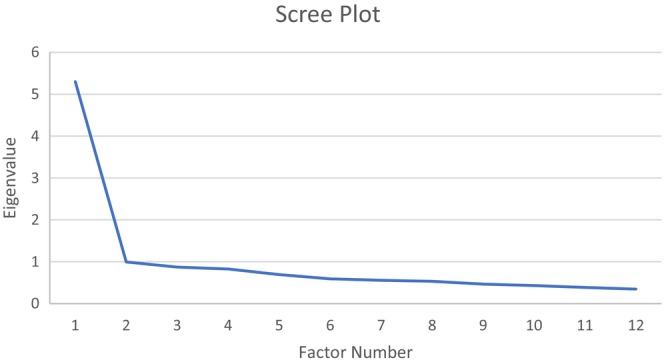
Scree plot for the exploratory factor analysis of the I.ROC.

- Step 1: KMO and Bartlett's test was significant (0.92; p = <0.001). Item correlations showed that all items correlated sufficiently.
- Step 2: The extraction method Maximum Likelihood revealed two factors with an eigenvalue = 1 explaining 52.5% of the variance. The two‐factor solution was confirmed by the scree plot (see Figure 1). That factor solution was extracted again with the Maximum Likelihood method, now with an Oblimin rotation to learn how to best allocate the items among the factors. Factor loadings of items ranged from 0.37 to 0.77 (Table 5).
- Step 3: The research team agreed on item distribution for 11 items. Item 2 (Life Skills) loaded equally on both factors. The research team decided to locate this item to Factor 2 because its content most relates to the other items in this factor.
- Step 4: For both factors, all inter‐item correlations were *r* < 0.6.
- Step 5: The internal consistency reliability for the total score on the 12‐item scale (mean = 3.39, sd = 0.088) was good: α = 0.88. Internal consistency reliability of factor 1 (α = 0.86) was good as well, but for factor 2 (α = 0.67) reliability was not sufficient.
- Step 6: After deliberation among the research team the factors were labelled, Factor 1 ‘Empowerment’ and Factor 2 ‘Vitality and activity’.

**TABLE 5:** Factor loadings from the exploratory factor analysis of I.ROC.

Structure matrix I.ROC		Factor	
Item number of questionnaire	Description	Empowerment	Vitality and activity
1	Mental health	**0.757**	0.537
2	Life skills	0.563	**0.563**
3	Safety and comfort	**0.588**	0.404
4	Physical health	0.612	**0.675**
5	Exercise and activity	0.413	**0.703**
6	Purpose and direction	**0.659**	0.579
7	Personal network	**0.525**	0.423
8	Social network	0.464	**0.551**
9	Valuing myself	**0.740**	0.465
10	Participation and control	**0.624**	0.395
11	Self‐management	**0.765**	0.487
12	Hope for the future	**0.754**	0.440

*Note:* Extraction method maximum likelihood method. Rotation method: Oblimin with Kaiser normalisation. In bold: items belonging to the subscale.

In a secondary analysis we analysed the data, while making a distinction between two groups: patients with Severe Mental Illness (SMI, *N* = 858) and common mental disorders (non‐SMI, *N* = 3011). Separate factor analyses for these two groups resulted in the same factor structure, only item two loaded slightly differently between the two groups. This item loaded in the non‐SMI group somewhat higher on the factor ‘Vitality and activity’ (0.528) compared to the factor ‘Empowerment’ (0.507), which confirmed the earlier classification of this item by the research team.

### 3.3 Confirmatory Factor Analysis

We investigated the factor structure in the randomly selected second half of the data (sample *N* = 4340) with CFA. We tested the fit of the 12 items with a single factor model, the 4‐factor model (HOPE structure), three 2‐factor models (Monger et al. 2013; Dickens et al. 2017; van Druten et al. 2024) and the EFA model from the present study (as was found for the first half of the dataset with EFA). For all models, modification indices were inspected. Allowing for correlation between the error terms of items 10 and 11 (Participation & Control and Self‐management) improved the fit for all models except for the 4‐factor model. We also present the fit of these modified models in Table 6.

**TABLE 6:** Confirmatory factor analysis of I.ROC total population (*N* = 4340).

Model	χ^2^	df	χ^2^/df	CFI	NFI	TLI	SRMR	RMSEA	CI90
Single factor	1816.37*	54	33.64	0.910	0.908	0.890	0.046	0.087	0.083–0.090
Modified	1464.67*	53	27.64	0.928	0.926	0.910	0.042	0.078	0.075–0.082
4 factor model	1457.74*	48	30.37	0.928	0.926	0.901	0.040	0.082	0.079–0.086
2 factor (Monger)	1800.29*	53	33.97	0.911	0.909	0.889	0.045	0.087	0.084–0.091
Modified	1459.59*	52	28.07	0.928	0.926	0.909	0.042	0.079	0.076–0.083
2 factor (Dickens)	1568.73*	53	29.60	0.923	0.92	0.904	0.042	0.081	0.078–0.085
Modified	**1261.05** *	**52**	**24.25**	**0.938**	**0.936**	**0.922**	**0.039**	**0.073**	**0.070–0.077**
2 factor (Van Druten)	1466.73*	53	27.67	0.928	0.925	0.910	0.041	0.079	0.075–0.082
Modified	**1144.61** *	**52**	**22.01**	**0.944**	**0.942**	**0.929**	**0.038**	**0.070**	**0.066–0.073**
2 factor (present EFA)	1537.45*	53	29.01	0.924	0.922	0.906	0.043	0.080	0.077–0.084
Modified	**1222.25** *	**52**	**23.50**	**0.940**	**0.938**	**0.924**	**0.040**	**0.072**	**0.069–0.076**

*Note:* The baseline χ^2^ = 19675.6. Bold values indicate statistical significance at *p* < 0.001.

* *p* < 0.001.

The best fit was found for the van Druten et al. (2024) model and for the model resulting from the EFA on the first half of the dataset of the present study, allowing for correlation between error terms for item 10 and 11, and almost reached the cut‐off of good fit of CFI = 0.95. However, the differences in fit with the other models were marginal and confidence intervals for RMSEA overlapped.

Again, we re‐analysed the data, separately for the two groups previously distinguished: patients with SMI (*N* = 843) and non‐SMI (*N* = 3045).

As shown in Table 7, for the subgroup with common mental disorders (non‐SMI), the model proposed by van Druten et al. (2024) (with modification allowing for correlation between error terms for items 10 and 11) fitted best of all. The model based on EFA of the present study (the other half of the dataset) with modification fitted second best.

**TABLE 7:** Confirmatory factor analysis of I.ROC non SMI (*N* = 3045).

Model	χ^2^	df	χ^2^/df	CFI	NFI	TLI	SRMR	RMSEA	CI90
Single factor	1311.01*	54	24.28	0.904	0.900	0.882	0.047	0.088	0.084–0.092
Modified	1029.43*	52	19.80	0.925	0.921	0.905	0.043	0.079	0.075–0.083
4 factor model	1036.39*	48	21.59	0.924	0.921	0.896	0.042	0.083	0.079–0.087
2 factor (Monger)	1290.12*	53	24.34	0.905	0.902	0.882	0.047	0.088	0.084–0.092
Modified	1016.02*	51	19.92	0.926	0.922	0.904	0.043	0.079	0.075–0.084
2 factor (Dickens)	1130.78*	53	21.34	0.917	0.914	0.897	0.044	0.082	0.078–0.087
Modified	**868.24** *	**51**	**17.02**	**0.937**	**0.934**	**0.919**	**0.040**	**0.073**	**0.069–0.077**
2 factor (Van Druten)	1031.46*	53	19.46	0.925	0.921	0.907	0.042	0.078	0.074–0.083
Modified	**763.12** *	**51**	**14.96**	**0.945**	**0.942**	**0.929**	**0.038**	**0.068**	**0.064–0.073**
2 factor (present EPA)	1100.86*	53	20.77	0.920	0.916	0.900	0.044	0.081	0.077–0.085
Modified	**834.69** *	**51**	**16.37**	**0.940**	**0.936**	**0.922**	**0.040**	**0.072**	**0.067–0.076**

*Note:* Baseline model: I^2^ = 13101.33*. Bold values indicate statistical significance at *p* < 0.001.

* *p* < 0.001.

We repeated the analyses for the *N* = 843 patients with Severe Mental Illness. Results are shown in Table 8.

**TABLE 8:** Confirmatory Factor analysis of I.ROC_SMI (*N* = 843).

Model	χ^2^	df	χ^2^/df	CFI	NFI	TLI	SRMR	RMSEA	CI90
Single factor	372.71*	54	6.90	0.915	0.903	0.896	0.048	0.084	0.076–0.092
Modified	289.51*	52	5.57	0.937	0.924	0.920	0.043	0.074	0.066–0.082
4 factor model	298.62*	48	6.22	0.933	0.922	0.908	0.042	0.079	0.071–0.088
2 factor (Monger)	372.21*	53	7.02	0.915	0.903	0.894	0.047	0.085	0.077–0.093
Modified	289.49*	51	5.68	0.937	0.924	0.918	0.043	0.075	0.067–0.083
2 factor (Dickens)	322.74*	53	6.09	0.928	0.916	0.911	0.045	0.078	0.070–0.086
Modified	**246.38** *	**51**	**4.83**	**0.948**	**0.936**	**0.933**	**0.040**	**0.068**	**0.059–0.076**
2 factor (Van Druten)	321.48*	53	6.07	0.929	0.916	0.911	0.044	0.078	0.070–0.086
Modified	**242.91** *	**51**	**4.76**	**0.949**	**0.937**	**0.934**	**0.040**	**0.067**	**0.059–0.076**
2 factor (present EPA)	310.09*	53	5.85	0.932	0.919	0.915	0.044	0.076	0.068–0.084
Modified	**233.97** *	**51**	**4.59**	**0.951**	**0.939**	**0.937**	**0.040**	**0.066**	**0.057–0.074**

*Note:* Baseline model: I^2^ = 3827.21*. Bold values indicate statistical significance at *p* < 0.001.

* *p* < 0.001.

Overall, the fit of the models is slightly better in the data from patients with SMI compared to non‐SMI patients with common mental disorders. Also, here we found that allowing for correlation between error terms of item 10 and 11 improved the fit consistently for all models.

The EFA two‐factor solution of the present study had the best fit for the SMI‐group, closely followed by the van Druten et al. (2024) model and the Dickens et al. (2017) model. However, the HOPE and the single factor solution were quite close to a good fit as well.

## 4 Discussion

This study focused on the structural validity of the Individual Recovery Outcomes Counter (I.ROC) and was investigated using data from a broad group of mental health care clients treated in different health care settings in five mental health care organisations across the Netherlands. The I.ROC is a user‐friendly and visual tool to measure the client's perspective on the broad concept of recovery, that is, personal, social, and symptomatic recovery (Ion et al. 2013; Monger et al. 2013). This is relevant because in mental health care, nowadays it has become more important not only to measure symptoms but also to monitor strength‐based recovery in various life areas of clients (van Weeghel et al. 2019; Llewellyn‐Beardsley et al. 2019; Dell et al. 2021; Baufeldt and Dawson 2022). Although previous international studies showed the good psychometric qualities of the I.ROC, results about the factor structure diverged, and thus there is no consensus about the subscales of the instrument (Monger et al. 2013; Dickens et al. 2017; Rudd et al. 2020; Beckers et al. 2022; Sportel et al. 2023; van Druten et al. 2024; Garrido‐Cervera et al. 2025).

The exploratory factor analysis (*n* = 4295) revealed a two‐factor structure: ‘Empowerment’ (8 items) and ‘Vitality and Activity’ (4 items). This factor structure shows the most similarity with the two factor structure found by (Dickens et al. 2017), where ‘life skills’ and ‘purpose and direction’ are reversed and belong to another factor. However, the exploratory factor structure of this study bears little resemblance to factor structures found in other Scottish (Monger et al. 2013; Rudd et al. 2020), Spanish (Garrido‐Cervera et al. 2025) and Dutch research (van Druten et al. 2024). The internal reliability for Factor 1 ‘Empowerment’ was good (*α* = 0.86). However, reliability of the second factor ‘Vitality and Activity’ was questionable (*α* = 0.67). Indeed, the accepted guideline for the Cronbach's alpha is between 0.70 and 0.90 (de Vet et al. 2011). Moreover, the internal reliability of the total scale of the I.ROC was also good (*α* = 0.88).

Confirmatory factor analyses (*N* = 4340) showed that the two‐factor model found in a general Dutch population (van Druten et al. 2024) had the best fit (CFI = 0.944), followed by the two‐factor solution of the present study (CFI = 0.940). Although with slightly lower CFI values (CFI ≥ 0.910), the one, two, three, and four factor structures found in Scottish (Monger et al. 2013; Dickens et al. 2017; Rudd et al. 2020), Spanish (Garrido‐Cervera et al. 2025) and other Dutch research (Beckers et al. 2022; Sportel et al. 2023) were also supported with indices that showed sufficient fit. As several factor solutions demonstrated acceptable fit, no one model emerged as definitively superior. This suggests, as the broad definition of recovery implies (van Weeghel et al. 2019; Llewellyn‐Beardsley et al. 2019; Dell et al. 2021; Baufeldt and Dawson 2022), that this concept is multi‐faceted and cannot be easily captured in a uniform, dominant factor structure.

Secondary analyses for the separate groups non‐SMI and SMI revealed that the two‐factor structure of van Druten et al. (2024) fitted best within the non‐SMI group (CFI = 0.945) and the two‐factor structure found in this study matched best with the SMI group (CFI = 0.951). This could be explained by the fact that the characteristics of the less severe (non‐SMI) group are probably more similar to the general population as compared to SMI. Although the factor models match slightly better in the SMI subgroup, the CFIs of all factor structures in both subgroups (SMI and non‐SMI) reveal similar good fits (≥ 0.904).

### 4.1 Strengths and Limitations

A strength of this study is that we had access to a large dataset (*N* = 8635) from five Dutch mental health care organisations which consists of I.ROC data from a heterogenous patient group (SMI and non‐SMI as well) treated in various settings which was representative of the population treated in Dutch mental health care. The large dataset complies amply with the requirements for the minimum needed sample size to perform factor analyses, that is, 10 patients per item with a minimum of 100 patients (de Vet et al. 2011). There was also sufficient data to perform exploratory and confirmatory factor analyses on two separate randomly selected datasets.

A limitation is the cross‐sectional design of this study. The stability of the found factor structure (and possible factorial invariance) over a longer period of time was not investigated. Therefore, further research with longitudinal data is recommended. Furthermore, we classified patients in the SMI or non‐SMI group based on the treatment setting, because this was a feasible approach and came closest to the consensus definition of SMI (Delespaul 2013). However, we are not sure if a limited group of patients that we currently classified in the non‐SMI group possibly would be assigned to the SMI group if we were able to apply the consensus definition on the individual patient data.

### 4.2 Clinical Implications

Up to now no research has revealed a unified factor structure of the I.ROC. This might be explained from three perspectives.

First, this could be due to the translation from English into Dutch and the fact that there are cultural differences between Scotland and the Netherlands and among the target groups included in the various studies. Because the interpretation and meaning of the items could be different due to language, culture, and background, the factor structure also may vary between countries and target groups (de Vet et al. 2011). This would suggest that we should use the factor structures found in research of the own country where the (most) similar target group was included. However, we think that translation and culture might not be the only explanation for the differences in factor structures.

A second plausible perspective for the fact that no clear dimensions can be distinguished could be that the I.ROC was developed from a clinimetric rather than a psychometric approach. The first approach is practical and outcome‐oriented and aims to assess multiple attributes of treatment outcomes with a single index. In contrast, the psychometric approach is more theoretically driven and aims to measure one or more clear constructs, for example types of psychopathology such as depression and anxiety (Fayers and Hand 2002). Thus, the clinimetric approach to the development of the I.ROC, and also the broad formulation of the questions with high correlations among them, might have hindered the discovery of an underlying factor structure and makes such a structure of distinct factors probably less important for the I.ROC.

But what does this mean for the way the I.ROC should be used in practice? In any case, the I.ROC can be used as a set of 12 life domains depicting well‐being and recovery. The total score (one factor structure) gives an overall picture of well‐being and showed good internal consistencies (*α* > 0.8) in various researches (Dickens et al. 2017; Beckers et al. 2022; Sportel et al. 2023).

With some caution, the subscales based on factor solutions found in this or previous research can be used. Then it is important to choose the factor structure with the best match with the own population. Thus, for Dutch mental health care the factor structures of van Druten et al. (2024) and the present study can be used. An advantage of the two factor structure found in this study that is, ‘Empowerment’ and ‘Vitality & Activity’ is that these concepts are more clear and comprehensible compared to the broad dimensions found by van Druten et al. (2024) that is, ‘Wellbeing, control, network and meaningfulness’ and ‘Health safety and abilities’.

For the interpretation at the client level, the dialogue between client and practitioner about the results remains important and discussion of the item scores visualised in a spider's web is also well applicable (Ion et al. 2013; Monger et al. 2013). In addition to the total scale, at group level, the subscales could be complementary for a summarised overview (Sportel et al. 2023), provided the cronbach's alphas of the subscales are sufficient. Therefore, before using the subscales at group level, we recommend calculating the cronbach's alphas in the corresponding dataset first and using the subscales only if *α* ≥ 0.70.

## 5 Conclusion

We can conclude that the total score of the I.ROC appears a good indicator of the overall level of recovery and wellbeing of a client and close inspection of the scores on the 12 individual items may reveal (further) goals or targets for treatment. Various research indicated no unified factor structure for the I.ROC, probably because of cultural invariance, the clinimetric approach and the broad formulation of I.ROC questions. In this study we found confirmation for two‐two factor structures (1: ‘Empowerment’ and ‘Vitality and Activity’ and 2: ‘Wellbeing, control, network and meaningfulness’ and ‘Health safety and abilities’). Alternative two‐, three‐, and four‐factor models proposed in other studies also demonstrated an acceptable fit. To investigate the stability of the found factor structures over a longer period of time, further research with longitudinal data is recommended.

## 6 Relevance for Clinical Practice

With the emergence of the broad and strength‐oriented view on recovery in mental health care, a need has arisen for measurement instruments assessing clients' (progress in) recovery. The I.ROC is such a recovery‐oriented measure that is increasingly used in Dutch mental health care. The I.ROC identifies recovery goals in various areas of life and is suitable for monitoring progress. The I.ROC supports the client and practitioner in setting and evaluating these goals and can also be used by teams to learn from I.ROC outcomes at group level.

For the dialogue between client and practitioner about the life domains important to recovery, the 12 individual items visualised in a spider diagram are well applicable. Moreover, the total I.ROC score of 12 items gives an overall and global picture of the clients' well‐being. In addition, findings of this study show that the two factor structures found in Dutch populations can be helpful to get a summarised view on the data, especially to monitor trends at group level. Before using these two factor structures, it would be recommended to calculate the Cronbach's alphas in the corresponding dataset to confirm that they meet the minimum level of 0.7.

## Author Contributions

M.M. and E.B. wrote the study proposal in cooperation with F.S., G.S., N.W., and E.G. M.M. led the research project. F.S. and M.M. managed the data submission. F.S. and E.B. conducted the factor analyses in consultation with M.M., G.S., N.W. M.M., G.S., and E.B. wrote the manuscript. All authors provided comments on manuscript drafts and approved the final manuscript.

## Ethics Statement

This study was conducted in accordance with the Declaration of Helsinki and the General Data Protection Regulation (GDPR). Each organisation received approval for data submission from their own local Scientific Review Board, which confirmed this research was performed with anonymous data and the Medical Research Involving Human Subjects Act (WMO) did not apply to this study. The data that support the findings of this study are available from the corresponding author M.M., anonymous, and upon reasonable request. The authors declare no competing interests.

## Conflicts of Interest

The authors declare no conflicts of interest.

## Data Availability

The data that support the findings of this study are available on request from the corresponding author. The data are not publicly available due to privacy or ethical restrictions.
